# The role of the University of Zagreb School of Medicine in the development of education, health care, and science in Croatia

**DOI:** 10.3325/cmj.2018.59.185

**Published:** 2018-10

**Authors:** Marijan Klarica

**Affiliations:** Dean of the University of Zagreb School of Medicine, Zagreb, Croatia *marijan.klarica@mef.hr*

**Figure 1:**
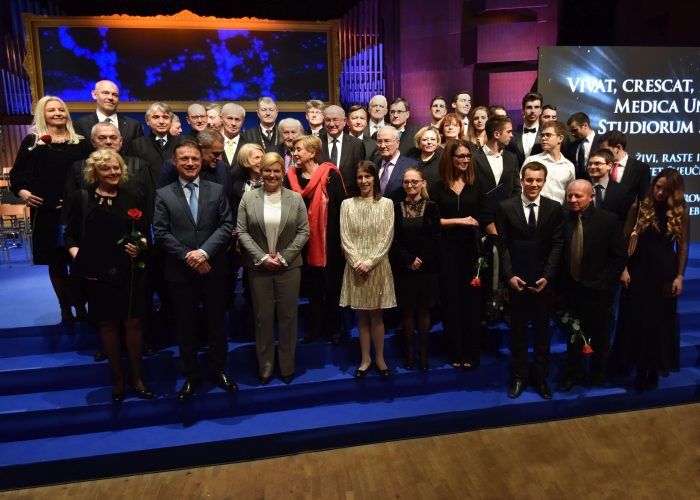
Highest state dignitaries, faculty members, recipients of awards, and guests at the celebratory meeting of the School Council in the Vatroslav Lisinski concert hall on December 17, 2017, for the 100th anniversary of the University of Zagreb School of Medicine.

The University of Zagreb School of Medicine (UZSM) was founded by the Croatian Parliament in 1917, and we can proudly say that it is one of the oldest medical schools in this part of Europe. The School’s hundredth anniversary was commemorated through a variety of events and projects (symposia, exhibitions, concerts, a monograph, and a documentary movie). However, the key event was the celebratory meeting of the School Council in the “Vatroslav Lisinski” concert hall on December 17, 2017, attended by the highest state dignitaries and many guests from our Croatian and foreign partner institutions (Figure 1).

Ever since its foundation, the School of Medicine has played a key role in the development of biomedicine in Croatia and the region. It founded all other University Schools of Medicine in Croatia and many of those in neighboring countries (1). It also founded first graduate programs in nursing, Zagreb University School of Dental Medicine, Faculty of Education Sciences, Faculty of Kinesiology, etc (1). Today, the school is the one of the leading constituents of University of Zagreb in terms of size, scientific output, and teaching quality.

The School has around 800 employees, with 1300 external associates involved in teaching of 2800 students enrolled in different teaching programs (around 1900 students at the Integrated Studies in Medicine in Croatian, 350 at the Integrated Studies in Medicine in English, 130 at the University Graduate Studies in Nursing, and 150-500 at PhD and specialist studies) (1). Integrated studies (5581 teaching hours) are organized through 34 Chairs, six Course Councils, and four Cabinets, located not only at the School's main premises, but also at “Andrija Štampar” School of Public Health, Clinic for Infectious Diseases “Dr Fran Mihaljević,” and other clinics, community health centers, and public health institutes.

Acquiring clinical skills is not possible without working with real patients and bedside teaching, which is why the School organizes clinical teaching at 45 clinics and 14 clinical departments at University Hospitals (UH) Dubrava, UH Center Zagreb as the biggest and most important teaching base, UH Merkur, UH *Sestre Milosrdnice* with all its constituents, including Klaićeva Children's Hospital, UH Sveti Duh, and University Psychiatric Clinic Vrapče. Clinics and clinical departments of UZSM encompass 139 Referent Centers of the Ministry of Health. In these Centers, experts from the School convey the most up-to-date knowledge on prevention, diagnostics, and treatment to young physicians and residents using the state-of-the-art technology. Our teaching base would not be complete without the help of 27 partner institutions – general hospitals and health care institutions from all over Croatia (from Vukovar to Dubrovnik) (1).

Over the years, UZSM has educated numerous generations of Croatian physicians and nurses, thus creating immeasurable value for the citizens of Croatia. So far, 24 841 students have attained the title of doctor of medicine (1), 3672 attained the master’s degree, and 2566 attained their PhD degree (1). The School also holds the leading position in Croatia when it comes to the introduction and accreditation of new specialist studies. We have accredited 46 specialist studies, and at the end of the accreditation process, we will be the only school in Croatia with all 48 specialist medical studies required by the Ministry of Health and Social Welfare. Besides, we have accredited six more specialist studies outside of the regular program of specialist education. Worthy of especial mention are several European Centers for Subspecialist Education that function within our institution. These centers provide subspecialist education to residents from Europe and around the world and issue a European certificate.

From the very beginnings, the School’s teachers from all biomedical fields have complemented their teaching by publishing textbooks and manuals. Today, each Chair has its own set of textbooks, which follows the teaching plan and program. Textbook publishing has increased over time, so today the faculty publishes 30-40 textbooks and manuals a year, that is, almost as many as three a month. Textbooks are published not only in Croatian, but also in many other languages. In this way, our teachers have contributed to the creation of Croatian medical terminology, enriching the Croatian language and national scientific heritage.

Our institution has always been at the fore when it comes to internationalization of academic and scientific work. We were the first to establish the Integrated Studies in English and have directed many efforts toward fostering mobility. Each year there is an increase in the number of UZSM students and teachers who spend some time in academic centers abroad and foreign students and teachers who come to Zagreb. Our School has contributed to the development of doctoral studies in Europe and the organization of international conferences on harmonization of study programs. We received the Certificate for Quality Assurance in the European higher education area in 2013 and the CeQuint Certificate for Quality in Internationalization in 2015.

Owing predominantly to the scientific production at our School, Croatian medicine remains among the most successful and highly internationally recognized segments of Croatian science. According to the number of scientific articles published in international journals, it is the leading scientific institution in Croatia. Almost every fourth article from scientific institutions in Croatia is authored by members of our faculty, which shows that we considerably contribute to the positioning of University of Zagreb in the top 3% of world universities with greatest scientific output (Table 1). According to the SCOPUS database, at least one author per 34.7% scientific articles from the University of Zagreb is from UZSM (2).

**Table 1:** The total number of articles in the SCOPUS database from five leading institutions in Croatia (2)

Affiliation name	Documents		City	Country/Territory
	Affiliation	Institution		
University of Zagreb	44077	**62948**	Zagreb	Croatia
University of Zagreb School of Medicine	21823	**21844**	Zagreb	Croatia
Institute Ruđer Bošković	16362	**16362**	Zagreb	Croatia
University of Split	7850	**7943**	Split	Croatia
University of Rijeka	6103	**6565**	Rijeka	Croatia

Biomedicine (*key words in database SCOPUS: Medicine; Biochemistry, Genetics and Molecular Biology; Pharmacology, Toxicology and Pharmaceutics; Immunology and Microbiology; Neuroscience; Veterinary; Psychology; Health Professions; Dentistry) accounts for 41.8% of the total scientific production from Croatia, and 47.3% of articles from the field of biomedicine had at least one author from UZSM (2). A clear insight into the extent of biomedical scientific production from our School can be gained from the Leiden list. Leiden Ranking is a list of universities that published at least 1000 articles in the Web of Science in the previous four years, and only according to the more “serious” indexes available there (Science Citation Index Expanded – SCI-EXPANDED, Social Sciences Citation Index-SSCI, Arts & Humanities Citation Index-A&HCI) (3). The University of Zagreb is ranked 417th out of 938 world universities that meet the listed criteria. In the “Biomedical and Health Sciences” section, the University of Zagreb is ranked 396th (3).

According to the Scopus database, in the last few years scientists from our institution have published between 1100 and 1200 articles a year (100 articles a month) (2). In the Web of Science, they have published 400 articles per year (around 120 articles in Q1), 10% of which (more than 40) are published in journals with the highest impact factor, which speaks for itself when it comes to research quality at our institution (4) (Table 1).

Data available in SCOPUS database show that 21.3% of all scientific articles from Croatia were authored by at least one researcher from the UZSM. Since 2008, the percentage has decreased to 19.8% due to the increasing productivity of other institutions (2).

Table 2 shows the current intense scientific activity of UZSM employees, which presents a solid basis for the education of PhD students (there are three doctoral studies: Biomedicine and Health, in Croatian and English, and Neuroscience). We take great pride in the fact that the School of Medicine has established two centers of excellence – the Center of Excellence for Reproductive and Regenerative Medicine and the Center of Excellence in Basic, Clinical and Translational Neuroscience. These centers have successfully established a multidisciplinary collaboration with the majority of the University of Zagreb constituents, various Croatian scientific institutions, and renowned scientific institutions throughout the world.

**Table 2:** Active scientific research projects at the University of Zagreb School of Medicine on October 16, 2018

Program	Number of projects	Total allocated to UZSM	Total per project
Horizon 2020	6	1 896 562.50 €	21 059 118.50 €
European Cooperation in Science and Technology (COST) Action	1	no financing	
German Academic Exchange Service (DAAD)	4	no financing	
Croatian Science Foundation	29	27 352 648.11 kn	
ADRIS	1	200 000.00 kn	200 000.00 kn
University of Zagreb financial support, 2018	111	2 760 000.00 kn	
EU – European Social Fund (EXPPAND):	1	1 756 562.71 kn	
Centers of Research Excellence in Croatia (ZCI)	2	74 000 000.00 kn	
BICRO-HAMAG, Croatian Agency for SMEs, Innovations and Investments*	2		

*Financial support to the technology transfer centers; administratively managed by the university.

UZSM teachers are well-known Croatian and international experts and heads of prominent Croatian institutions. If the Latin proverb “*Gloria discipuli, gloria magistri“* is true and the glory of the students is indeed the glory of their teachers, then we can proudly say that the leading positions of our former students in the world's healthcare and scientific institutions corroborate the value and quality of education at our School. Many of our teachers lead international professional organizations, organize international conferences, participate in international panels and committees for evaluation of research projects, work as editors in world's leading journals, and are the recipients of the national, European, and world awards. Among many achievements of our faculty, we are especially proud of the International Health Day, since on April 7, 1948 the president of the World Health Organization Assembly was one of the School's teachers and deans, Andrija Štampar.

The School was faced with one of its greatest historical challenges during the Croatian War for Independence in the 1990s. The Government reacted to the threat of war by organizing the Main Medical Headquarters of the Republic of Croatia, which mainly included our faculty members, headed by professors Andrija Hebrang and Ivica Kostović. The Headquarters was the first state defense organization that utilized both civilian and military infrastructure in the setup of an integrated health system closely related to the School (5). Bearing in mind the proportion of the humanitarian crisis of that time (huge numbers of wounded, refugees, and displaced persons, obstruction of humanitarian help, missing person and prisoner registers, prisoner exchange, identification of postmortem remains, etc), we can boast of the lowest mortality among the wounded in war literature, amounting to a mere 1.4% (5). In addition, not a single epidemic or massive food poisoning occurred, and only ten cases of tetanus infections were recorded. With more than 600 000 refugees, the number of infectious diseases was smaller than in peacetime conditions (5). Our institution has played a leading role in the process of identification of postmortem remains (more than 80% successful identifications) and in forensic witnessing related to the search for missing soldiers and civilians. The activity of the School during the War of Independence resulted in 371 articles published in Croatian and 308 articles published in international journals.

The high scientific activity of the faculty necessarily led to the foundation of the School’s own journals. *Acta Facultatis Medicae Zagrebiensis,* established in 1953 was regularly coming out by 1992, when it was replaced by the *Croatian Medical Journal (CMJ)*. The *CMJ*, which is a joint publication of three Schools of Medicine in Croatia (Zagreb, Rijeka, Split), soon gained considerable international visibility. Since one of the Journal’s main tasks is to promote Croatian biomedicine, members of the faculty were invited to present their recent work in a special thematic issue to honor the School’s 100th anniversary. The nine articles published in the current issue represent only a small fraction of the School's entire research output; however, the articles underwent the peer review process in a relatively short period and were selected for their valuable contribution to their respective fields at the international level.

**Figure 2:**
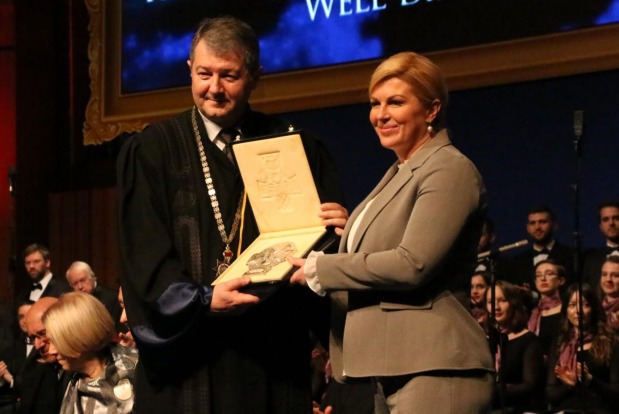
The President of the Republic of Croatia Kolinda Grabar-Kitarović awarded the University of Zagreb School of Medicine with the Croatian National Award for the outstanding contribution to education and research, social and humanitarian activities, and continuous health care advancement. The photograph shows the President handing out the Award to the Dean, Professor Marijan Klarica.

In honor of its hundredth anniversary, on July 31, 2017, the President of the Republic of Croatia awarded the School with the Croatian National Award for outstanding contribution to education and research, social and humanitarian activities, and continuous health care advancement (Figure 2). There is nothing more to add but once again wish the School a happy anniversary by saying: “*Vivat, crescat, floreat Facultas Medica Universitatis Studiorum Zagrabiensis*!”
